# Pilot Research as Advocacy: The Case of Sayana Press in Kinshasa, Democratic Republic of the Congo

**DOI:** 10.9745/GHSP-D-16-00236

**Published:** 2016-12-23

**Authors:** Arsene Binanga, Jane T Bertrand

**Affiliations:** a Tulane International LLC, Kinshasa, Democratic Republic of the Congo.; b Tulane School of Public Health and Tropical Medicine, New Orleans, LA, USA.

## Abstract

In the Democratic Republic of the Congo (DRC), the Ministry of Health authorizes only physicians and nurses to give injections, with one exception—medical and nursing students may also give injections if supervised by a clinical instructor. The emergence of the injectable contraceptive Sayana Press in some African countries prompted the DRC to test the acceptability and feasibility of distributing Sayana Press and other contraceptive methods at the community level through medical and nursing students. Sayana Press is similar in formulation to the injectable contraceptive Depo-Provera but contains a lower dose and is administered subcutaneously using a single-use syringe with a short needle called the Uniject system. The Uniject system allows Sayana Press to be administered by community health workers without clinical training or by self-injection. In this pilot, the advocacy objective was to obtain approval from the Ministry of Health to allow medical and nursing students to inject Sayana Press, as a first step toward authorization for community health workers to provide the method. The pilot described in this article documents a process whereby an innovative approach moved from concept to implementation to replication in less than 2 years. It also paved the way for testing additional progressive strategies to increase access to contraception at the community level. Because the pilot project included a research component designed to assess benefits and challenges, it provided the means to introduce the new task-shifting approach, which might not have been approved otherwise. Key pilot activities included: (1) increasing awareness of Sayana Press among family planning stakeholders at a national conference on family planning, (2) enlisting the support of key decision makers in designing the pilot, (3) obtaining marketing authorization to distribute Sayana Press in the DRC, (4) implementing the pilot from July to December 2015, (5) conducting quantitative and qualitative studies to assess acceptability and feasibility, and (6) disseminating the findings to family planning stakeholders. Before the pilot, Sayana Press was relatively unknown in the DRC, and there was no precedent for medical and nursing students providing family planning methods or giving injections at the community level. In less than 12 months, the approach gained legitimacy and acceptance. The key Ministry of Health decision maker orchestrated the closing session of the dissemination meeting on next steps, paving the way for pilot tests of 3 new task-shifting approaches: insertion of Implanon NXT by medical and nursing students, self-injection for Sayana Press with supervision by students, and injection of Sayana Press by community health workers with no formal clinical training.

## Abstract (precis)

The pilot study obtained Ministry of Health approval to allow medical and nursing students to provide the injectable contraceptive Sayana Press and other methods in the community, paving the way for other task-shifting pilots including self-injection of Sayana Press with supervision by the students as well as injection by community health workers.

## BACKGROUND

The purpose of this article is to highlight the potential of pilot research studies to achieve advocacy objectives. Although the concept is not new, there is little in the published literature to indicate its use as a best practice in international family planning. Research is usually viewed as a means of generating relevant data on the topic, but this case study describes a pilot study that served as the catalyst to achieving change in regulations governing family planning service delivery in the Democratic Republic of the Congo (DRC). The pilot study used medical and nursing students to provide the injectable contraceptive Sayana Press (as well as other methods) as a means to increase family planning uptake and the modern contraceptive prevalence rate. Ultimately, the pilot study was designed to pave the way toward subsequent authorization for community health workers to provide Sayana Press.

Advocacy addresses different audiences at different levels. At the global level, family planning advocacy aims to increase investments from multilateral and bilateral donors as well as the private sector in a particular area (e.g., family planning in general or for a specific issue such as contraceptive security). Global advocacy efforts also aim to set health and development goals to which countries will aspire, as in the process surrounding the Sustainable Development Goals. At national and subnational levels, advocacy is frequently used to increase government commitment toward family planning objectives, such as a budget line item for contraceptive procurement or removal of tariffs on the import of contraceptives. It may also be directed to changes in policy or regulations that directly affect the delivery of family planning services, including task shifting to enable lower-level health workers such as nurses to perform clinical procedures previously reserved for physicians.^1^

The global health community increasingly considers advocacy an essential tool to influence financial and political decisions that support access to and use of voluntary high-quality family planning services. Family planning advocacy toolkits present guidelines for developing communication strategies and materials designed to influence policy decisions, including developing an advocacy strategy; engaging policy makers, health sector leaders, community leaders, and the private sector; working with the news media; and other resources.^2^ Best practices indicate the need to present reliable data that frame the issue in terms consistent with national priorities, while presenting the material in simple, easy-to-comprehend formats.

Advocacy generally employs a combination of evidence and emotional triggers. Advocates seek to gather and analyze existing data (e.g., Demographic and Health Surveys, Multiple Indicator Cluster Surveys, and other country-level studies) to inform their strategies, rather than generating their own data.^2^ Information alone, however, rarely achieves an advocacy objective. Qualitative research and the stories of those most affected by a specific policy or programmatic barrier usually complement quantitative data. Well-known and highly respected personalities can bring attention to an issue and deliver messages to a larger and wider audience (e.g., Angelina Jolie as a U.N. Ambassador). According to a 2006 survey in sub-Saharan Africa, respondents trusted faith-based organizations more than they trusted their own national governments; religious leaders are therefore uniquely positioned to reach both men and women to promote family planning and healthy reproductive behaviors.^3^

Effective advocacy efforts usually require quantitative data complemented by qualitative research and stories.

Despite available guidance on how to use family planning advocacy to achieve objectives, there is limited documentation on the results of these efforts. According to Smith and colleagues,^4^ no studies have specifically investigated decision makers' views on and use of family planning research and advocacy.

## THE ADVOCACY OBJECTIVE OF THE DRC PILOT STUDY

In early 2011 Sayana Press emerged as a promising means of increasing access to modern contraception at the community level in developing countries.^5^ Although its formulation (104 mg of depot medroxyprogesterone acetate per 0.65 mL dose) is similar to Depo-Provera, it contains a lower dose and is administered subcutaneously using a single-use syringe with a short needle called the Uniject system, which can be administered by trained community health workers and clients.

Sayana Press is similar to Depo-Provera, but contains a lower dose and is administered subcutaneously using a single-use syringe with a short needle, which can be administered by community health workers.

A World Health Organization (WHO) consultation in 2009 approved the use of injections by community health workers, even before Sayana Press became available,^6^ and successful pilots using Depo-Provera have been reported from other countries.^7^^–^^9^ Studies in Senegal and Uganda have explored acceptability and feasibility of introducing Sayana Press using community health workers,^10^ and a study in Ethiopia explored attitudes toward self-injection.^11^

In the DRC, a regulation limiting the provision of injections to only physicians and nurses represented a major barrier to community-level delivery of Sayana Press. An important exception, however, provided an open door to test an innovative approach—medical and nursing students are allowed to give injections if supervised by a clinical instructor. Thus, in the case study presented here, the advocacy objective was to obtain approval from the DRC Ministry of Health to distribute Sayana Press—a new method that was not currently part of the approved method mix—at the community level using medical and nursing students, as a first step toward subsequent testing and eventual authorization for community health workers to provide this method. Not only would this mechanism contribute to increasing access to this new method in the short term, it would also give future doctors and nurses a solid foundation in contraceptive technology and service delivery.

The first objective of the pilot was to obtain approval from the DRC Ministry of Health to allow medical and nursing students to distribute Sayana Press at the community level.

## PLANNING PHASE

Before the pilot began, a series of key activities paved the way to making it a reality: (1) a commitment to community-based distribution of contraception in the national strategic plan for family planning, (2) increased awareness of the new Sayana Press method among stakeholders, (3) the support of key decision makers in the design of the pilot, (4) legal authorization from the Ministry of Health to distribute Sayana Press, and (5) a donation of 60,000 doses from Pfizer through the United Nations Population Fund (UNFPA).

### Commitment to Community-Based Distribution

The Multisectoral Strategic Plan for Family Planning in the DRC: 2014–2020 identified community-based distribution as a key strategy for the country to accelerate achievement of its objective of 19% modern contraceptive prevalence use by 2020. This call for expansion of community-based distribution by the family planning stakeholder community was an important first step leading to the pilot.

### Increased Awareness of Sayana Press

In the months leading up to the pilot, Tulane University, the organization responsible for its implementation, sought opportunities to increase awareness of this new contraceptive method and the studies taking place in other sub-Saharan African countries. At the Third National Conference on Repositioning Family Planning in the DRC in December 2014, the researchers who also participated in organizing the conference seized the opportunity to widely diffuse information about Sayana Press and present the pilot experiences in other countries to the community of family planning stakeholders in the DRC. A physician from Senegal leading the Sayana Press initiative in that country gave an overview of the new contraceptive method at one of the early plenary sessions, and a UNFPA consultant working in Burkina Faso led a more clinically oriented session on the method. Seminars were organized in parallel with the conference for the local obstetrics and gynecology, nursing, and midwives societies to further disseminate information about this new method, including the ease of application by non-clinically trained personnel.

### Support of Key Decision Makers

Because of the regulation that only physicians and nurses can give injections in the DRC, it was unclear whether the Ministry of Health would give its approval to pilot test the use of medical and nursing students to give injections at the community level. It was therefore essential to enlist the support of the Ministry of Health, and in particular 2 departments (*Directions*) that had jurisdiction over the organizations involved in the pilot: the 10th Direction (*10ème Direction*) which oversees the National Program of Reproductive Health (Programme National la Santé de la Reproduction, or PNSR), and the 6th Direction (*6ème Direction*), which oversees the training institutes for nursing through the country.

The researchers obtained agreement from the director of the 10th Direction that he would chair a meeting of key stakeholders in January 2015. The objectives of the meeting were to (1) present the implementation and study design plans for the pilot introduction of Sayana Press; (2) solicit feedback from stakeholders; (3) encourage an open exchange of opinions on the benefits and challenges of this approach; and (4) obtain buy-in among family planning stakeholders for the pilot. Organizing the pilot as a research study that would assess the benefits and limitations of the approach enabled the decision makers to authorize this innovative approach to service delivery, but on a limited scale; further expansion of the approach would depend on the results of the research. Stakeholders were supportive overall and provided valuable feedback and opinions; however, they called for several changes to the plans, including the inclusion of both urban and rural health zones to make the results more generalizable for subsequent replication. At a follow-up meeting in February 2015, the director of the 10th Direction approved the research pilot.

Given that the research team intended to work through local medical and nursing schools, another key decision maker enlisted for support was the 6th Direction, which oversees nursing training institutes throughout the country. The proposed pilot was expected to appeal to the 6th Direction in several ways. Medical and nursing training institutes traditionally use a curriculum that focuses primarily on clinical care in hospitals and health facilities. The proposed activity would provide students with the experience of working at the community level, thus preparing them for a broader array of tasks in the future. Moreover, it would put students in direct contact with clients and enhance their skills in both counseling and service provision.

A local NGO, Association de Santé et Dévéloppement (Association for Health and Development), was hired to implement the pilot and entered into discussions with the director of the 6th Direction. The initial inquiries met with considerable enthusiasm, for the reasons noted above. The director's support was so enthusiastic that he offered the NGO affordable office space to oversee the initiative in the same building.

### Legal Authorization to Distribute Sayana Press

At a roundtable for government, donors, and partner organizations in December 2014 (in conjunction with the Third National Conference on Repositioning Family Planning in the DRC), the minister of health publicly announced a 1-year approval (also called a waiver) to allow the distribution of Sayana Press in the DRC. In that same month, the 3rd Direction (responsible for pharmaceutical products) issued the marketing authorization (known as AMM, *l'autorisation de mise sur le marché*) for a 12-month period. With this authorization in place, Pfizer donated 60,000 doses of Sayana Press in March 2015 for the pilot.

Critical milestones for the pilot included temporary legal authorization from the Ministry of Health allowing distribution of Sayana Press, marketing authorization for the method, and a donation of 60,000 doses from Pfizer.

## IMPLEMENTATION

In early 2015, 10 medical and nursing training institutes were selected to participate in the pilot. Each one nominated a member of its clinical faculty to serve as a focal point to supervise the students involved in the pilot. Members of the PNSR and several family planning implementing organizations developed the training curriculum and materials. In April and May 2015, 135 medical and nursing students received 7 days of training on multiple aspects of service delivery: contraceptive technology, management of side effects, eligibility and delivery of 4 methods (condoms, pills, CycleBeads, and Sayana Press), and procedures for referring interested clients to a nearby health center for clinical methods (e.g., intrauterine devices and implants). The students also participated in a 1-day field practicum, in which they gave family planning counseling, screened clients for eligibility, provided the 4 contraceptive methods to interested clients, and made referrals in a real-life community setting.^12^

10 medical and nursing training institutes were selected to participate in the pilot.

The pilot officially began in July 2015. The delivery of contraceptive methods took several forms: (1) campaign days, in which a group of approximately 15 to 20 medical and nursing students provided counseling and contraception to women from the community who had been informed of the opportunity to get free contraceptive services on a specific day; (2) house-to-house visits to counsel women and couples on the use of family planning (with delivery of methods to interested, eligible women); and (3) distribution of contraception on campuses or other sites in the community.^12^ The medical and nursing students were referred to as community-based distribution agents (*distributeurs à base communautaire*, or DBC). When given the choice of 4 methods available on-site and others available through referral to a nearby health facility, approximately one-quarter of clients chose Sayana Press on-site.

Service provision took several forms: through specific campaign days, house-to-house visits, and distribution on campuses or other community sites.

During the implementation of the pilot and related research, the researchers regularly updated the directors of the 6th and 10th Directions, but did not involve them directly in the routine operations of the pilot.

## KEY FINDINGS FROM THE RESEARCH COMPONENT

The research component of the pilot used mixed methods. The quantitative research consisted of 3 surveys: one among acceptors of Sayana Press (n = 374) who were interviewed directly after receiving Sayana Press, a second among 252 of the original 374 respondents at a 3-month follow-up, and a third among 124 of the 135 medical and nursing students who had participated, to assess their experience as community-based distributors. The qualitative component consisted of in-depth interviews with 29 key informants: Ministry of Health personnel in decision-making positions, the chief medical officers for selected health zones, nurses in fixed facilities, and staff from the organizations that implemented the pilot.

Key findings from the quantitative surveys and qualitative in-depth interviews are summarized below. Full results from the quantitative surveys will be published separately.

### Acceptors of Sayana Press

Among all Sayana Press acceptors, 51.6% had never used contraception, including traditional methods. Overall, their experience with Sayana Press was positive; 87.4% encountered no problems. Just over half (58.5%) felt some pain at the time of the injection, but only 9.7% reported pain afterward and 3.4% had side effects. Among acceptors who attended their follow-up appointment 3 months after the first injection, 92.3% received a second injection. The large majority was satisfied with the counseling and services received from the medical and nursing students.

Among all Sayana Press acceptors, about half had never used contraception, including traditional methods.

### Medical and Nursing Students

Six months after implementation began, 92% of students were still participating in the project. Of these, 46.8% were medical students and 53.2% were nursing students. The median age was 22 years old and most of the students (71.8%) were women. More than 90% reported that the community was favorable toward their services. The vast majority expressed satisfaction in serving as community-based distributors, and more than 95% would recommend it to others. Their primary complaint was lack of remuneration, followed by insufficient supervision and contraceptive stock-outs.

The vast majority of medical and nursing students expressed satisfaction in serving as community-based agents, and more than 95% would recommend it to others.

### Key Informants

Overall, key informants in decision-making positions (Ministry of Health personnel, chief medical officers for health zones, nurses, and staff from implementing organizations) responded positively to the pilot study and the strategy of using medical and nursing students as community-based distributors. They had not heard of opposition specifically directed toward Sayana Press or the pilot introduction, although there was a low level of opposition to family planning in general. Key informants stressed the need for careful training of community-based distributors. Zonal health authorities were also amenable to the community-based distribution method and were unaware of opposition at the community level. All favored expansion to other health zones, especially those that are heavily populated. The key informants cited several challenges: scheduling conflicts between students' academic program and the pilot, recurrent issues with contraceptive resupply of the community-based distributors, incomplete reporting of service statistics by the students on the distribution of products, and uneven responsiveness of the focal points in different training institutes. Despite these challenges, the staff involved in implementing the pilot were uniformly supportive of this method of using students to distribute contraception at the community level and encouraged its expansion to other training schools and other provinces.

Key decision makers in family planning were supportive of the pilot overall and favored its expansion.

## DISSEMINATION OF THE FINDINGS

In December 2015, the research team held a 1-day dissemination event at a hotel in Kinshasa with more than 80 participants. The audience for this event included the primary family planning stakeholders: representatives from the PNSR and the Programme National de Santé de l'Adolescent (National Program for Adolescent Health), other Ministry of Health authorities, family planning implementing organizations, military and police, faith-based-organizations, donors (e.g., U.S. Agency for International Development [USAID], UNFPA, WHO), and university researchers, among others.

The moderator was a well-known and highly respected figure in the local family planning community, which enabled the director of the 10th Direction to focus on presentations and provide commentary during the event. The program covered a series of topics: Sayana Press as a new method, details about the pilot implementation process, testimonials of focal points (supervisors) from several training institutes, and testimonials of 4 participating medical and nursing students. In later sessions, the research team presented highlights from the surveys of acceptors (on the day of the injection and 3 months later), the survey of students participating in the pilot, and a summary of the key informant interviews. From the tone of the discussion, the majority of the audience seemed amenable to the use of medical and nursing students to deliver Sayana Press.

A highlight of the advocacy process was in the final session of the dissemination event on next steps, led by the director of the 10th Direction. Rather than having the director or research team outline possible next steps, the director encouraged stakeholders to recommend possible variations for further testing. The audience collectively volunteered 17 approaches, for example, replicating the model in other provinces, using a similar approach in military and police health zones, having community-based workers (who receive short-term training to perform a specific task) deliver Sayana Press at the community level, piloting self-injection of Sayana Press, and conducting a similar pilot introduction of Implanon NXT (a contraceptive implant preloaded in a disposable applicator) by medical and nursing students, among others. Participants publicly endorsed the use of students as distributors of Sayana Press at the community level and called for the piloting of additional approaches that were very progressive by local standards. The director of the 10th Direction implicitly endorsed the pilot by encouraging the audience to recommend related pilots. Moreover, the final report of the research results for the pilot was issued with his signature.

At an event to disseminate the findings of the pilot, 80 key family planning stakeholders publicly endorsed the use of students as distributors of Sayana Press at the community level and called for replicating variations of the pilot.

**FIGURE 1:**
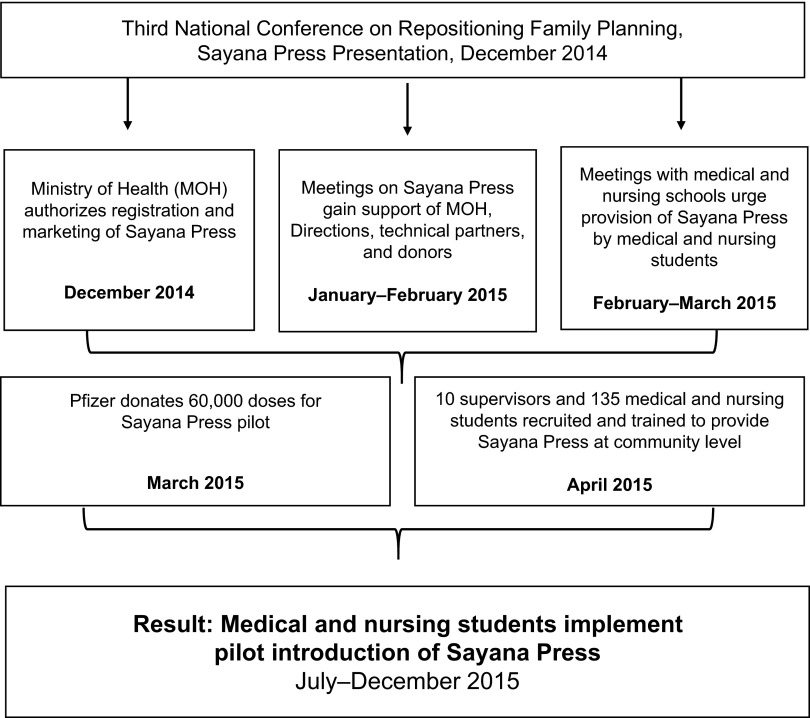
Steps Leading to the Democratic Republic of the Congo Government's Approval of Community-Based Provision of Sayana Press by Medical and Nursing Students, December 2014–December 2015

**FIGURE 2:**
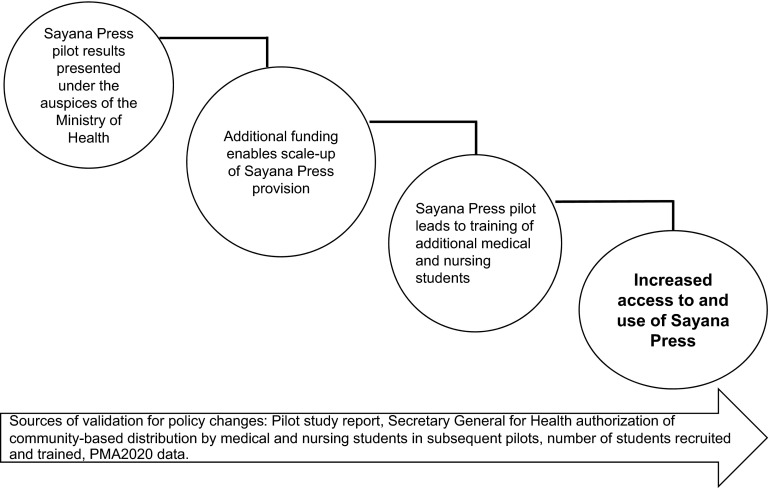
Pilot Program in the Democratic Republic of the Congo Prompts Policy Change and Increased Access to and Use of Sayana Press, 2015 Abbreviation: PMA2020, Performance Monitoring and Accountability 2020.

Figure 1 summarizes the sequence of steps that led to the achievement of the advocacy objective: the DRC government approval of community-based provision of Sayana Press by medical and nursing students. Figure 2 illustrates how this type of policy change influences access to contraception and increases contraceptive uptake.

## RAPID DIFFUSION, REPLICATION, AND TESTING

In early 2015 before the pilot, Sayana Press was relatively unknown, and there was no precedent for having medical and nursing students give injections at the community level. In less than a year, the approach gained legitimacy and acceptance. Both the 6th and 10th Directions were anxious to know what plans were under way to expand the use of students as community-based providers of Sayana Press (and other contraceptive methods) and when the next round of pilot introductions would begin. Within 12 months of the results dissemination, multiple activities were under way that built on the original pilot:

The pilot began with an innovative approach and moved from concept to implementation to replication in less than 2 years.

- **Institutionalizing the use of medical and nursing students within the 6th Direction.** A private donor came forward to fund the institutionalization of the approach through the 6th Direction, which will involve developing a more comprehensive module on contraceptive technology as part of preservice training and making community-level service provision a routine part of the students' training and as part of the health information system.
- **Replicating the approach in another province.** In October 2016, 119 nursing students received training as community-based distributors in Matadi, provincial capital of Kongo Central, and began providing Sayana Press, Implanon NXT, pills, male condoms, and CycleBeads at the community level.
- **Recruiting similar cadres of workers to distribute Sayana Press.** Several organizations funded by USAID recruited students and similar cadres (Red Cross workers) in other provinces to undertake community-level work in their projects.
- **Conducting additional pilot research projects**. Two new research pilots began in Kinshasa in late 2016 to test the use of medical and nursing students to (1) train women in self-injection of Sayana Press, and (2) insert Implanon NXT at the community level. A third pilot will begin in early 2017 to test the use of community health workers (who receive short-term training to perform specific tasks) to provide Sayana Press as part of an ongoing community-based distribution program. The Secretary General for Health authorized these 3 new pilots in a letter dated June 29, 2016 (N^o^MS.1251/SG/GM/1486/MK/2016).
- **Training medical and nursing students to deliver an expanded package of services.** A major donor came forward with additional funding to test the effectiveness of students in the provision of integrated maternal and child health and family planning services for first-time mothers ages 15 to 24. This gender-transformative project also incorporates the fathers of the babies as part of the population that would benefit from the intervention.

There were other positive outcomes from the Sayana Press pilot. The inclusion of the larger family planning stakeholder community in the initial deliberations over the pilot engendered support for and use of the final results. The positive findings from the pilot encouraged 2 major contraceptive donors—USAID and UNFPA—to procure larger quantities of the product to respond to the potential large demand for Sayana Press generated through other projects. The 2 social marketing projects based in Kinshasa also intensified their promotion of Sayana Press following the pilot.

Other outcomes of the pilot included the procurement of larger quantities of Sayana Press by 2 major contraceptive donors and intensified efforts among social marketing projects in Kinshasa to promote the method.

## KEY SUCCESS FACTORS

There is nothing novel in the concept of doing local research on issues that have been researched elsewhere as a means of obtaining local buy-in for innovative approaches. What is remarkable in this particular pilot is how fast the change took place. Although one cannot say with certainty what triggered the rapid change, several factors appear to have played a role.

First, the environment was ripe for innovation in the area of family planning. Since 2012, the DRC government has shown increasing political will toward family planning.^13^ The Prime Minister's Office has repeatedly linked the demographic dividend to the country's aspirations to be an emerging nation by 2030. The international donor community has reacted very favorably, both in terms of additional financial support to family planning initiatives and visibility in international fora (e.g., the invitation of the prime minister to address the closing plenary session at the 2016 International Conference on Family Planning in Nusa Dua, Indonesia.)^14^ The DRC has often lagged in development initiatives, as reflected by its high maternal and infant mortality rates. Yet in family planning, the DRC is emerging as a regional leader. The momentum around family planning in the DRC created an environment that was ripe for another progressive step in family planning: authorization of the distribution of Sayana Press at the community level.

Second, the research component of the pilot allowed for experimentation with the approach on a limited basis without requiring a large-scale policy change. Policy makers could reduce their political liability by withholding authorization on a larger scale, pending results of the pilot. If successful, they had evidence with which to support the expansion of the approach beyond the pilot sites. If unsuccessful, they could withhold approval, either entirely or pending modifications to the design.

## LESSONS LEARNED

Advocacy efforts require tailoring to specific countries because of differences in political, social, legal, and economic contexts. However, certain lessons from this experience in Kinshasa are likely applicable to other advocacy efforts:

1. There was clear political commitment to family planning and to community-based distribution as reflected in the National Multisectoral Strategic Plan for Family Planning: 2014–2020, which called for community-based distribution as a means to increase contraceptive access and thus increase the modern contraceptive prevalence rate to 19% by 2020.
2. A clear and achievable advocacy objective was set and informed by a group of influential and knowledgeable stakeholders.
3. Relevant decision makers were identified and enlisted from the start, not only to participate but to take a lead role in shaping the design of the research pilot.
4. The involvement of family planning stakeholders (including policy makers) in developing consensus on the design contributed to the success of this pilot and opened doors to next steps.
5. The research team cleared the necessary legal hurdles (obtaining authorization for the entry of Sayana Press into the local pharmaceutical market) with the support of local officials.
6. The pilot involved 3 Directions within the Ministry of Health, all of whom played a key role in its success.
7. The design lent itself to replication to other provinces and institutionalization within the Ministry of Health.^15^

## FINAL REFLECTIONS

We acknowledge that advocacy has limitations. It relies on a range of expertise to inform objectives and to implement policies and programs. Rarely do we have a counterfactual of what would have happened in the absence of the advocacy initiative. Moreover, serendipitous events can occur that either facilitate or hinder an advocacy effort. As a result, evaluation of the role of advocacy in improving health conditions may not be definitive. For example, Figure 2 points to plausible pathways by which advocacy influences behavioral outcomes among the target population, but it does not demonstrate cause and effect.

Curiously, the strength of this pilot was not in the precise findings it obtained but rather the process used in designing, implementing, researching, and disseminating the results publicly to a large group of relevant stakeholders. This being said, it is essential that the research methodology used to support advocacy objectives be of the highest quality, and that results—both positive and negative—be disseminated.

## Notes

**Peer Reviewed**

**First Published Online:** 2016 Dec 15
